# CXCL10 Involvement in Vestibular Migraine via the PI3K/AKT Signaling Pathway

**DOI:** 10.1111/cns.70655

**Published:** 2025-11-17

**Authors:** Mao‐mei Song, Ting‐yan Chen, Shi‐na Song, Ying‐jie Gao, Chang‐xin Li, Sui‐yi Xu

**Affiliations:** ^1^ Department of Neurology, Headache Center The First Hospital of Shanxi Medical University Taiyuan China; ^2^ Department of Neurology, Headache Center Shenzhen Nanshan People's Hospital and the 6th Affiliated Hospital of Shenzhen University Medical School Shenzhen China

**Keywords:** chemokine, CXCR3, dizziness, inflammatory cytokines, pain threshold, trigeminal nucleus caudalis, vertigo, vestibular nuclei

## Abstract

**Aims:**

C‐X‐C motif chemokine ligand 10 (CXCL10) is a member of the CXC chemokine family, known as a classical pain‐related chemokine. While CXCL10 is implicated in neuropathic pain, its role in vestibular migraine (VM) remains unclear.

**Methods:**

Serum CXCL10 levels correlated with dizziness and headache severity in VM patients. In the rat VM model, CXCL10, C‐X‐C motif chemokine receptor 3 (CXCR3), and phosphatidylinositol 4,5‐bisphosphate 3‐kinase (PI3K)/protein kinase B (AKT) signaling pathway expression were evaluated to explore the potential mechanisms of CXCL10 in VM.

**Results:**

Serum CXCL10 levels in VM patients were significantly elevated and positively correlated with headache and dizziness severity. In the rat VM model, CXCL10 and CXCR3 expression in the trigeminal nucleus caudalis and vestibular nuclei were significantly increased. Additionally, molecules related to the PI3K/AKT signaling pathway and downstream inflammatory factors showed significantly elevated expression.

**Conclusion:**

CXCL10 activates the PI3K/AKT signaling pathway, promoting the release of inflammatory factors, including interleukin‐1β, interleukin‐6, and tumor necrosis factor‐α, and is thus involved in the pathogenesis of VM.

## Graphical abstract

CXCL10 activates the PI3K/AKT signaling pathway, promoting the release of inflammatory factors, including IL‐1β, IL‐6, and TNF‐α, and is thus involved in the pathogenesis of VM.

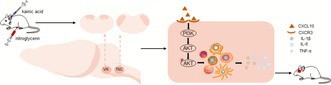

## 1 Introduction

Vestibular migraine (VM) is the leading cause of episodic vertigo in both adults and children [1]. Statistics indicate that VM accounts for approximately 11.6% of patients in dizziness clinics and 10.3% of those in migraine clinics [2, 3]. A retrospective analysis of VM research in low‐ and middle‐income countries included 26 studies, with 13 reporting prevalence rates ranging from 0.3% to 33.3% [4]. VM patients experience both migraine symptoms and vestibular dysfunction, such as dizziness. The frequent co‐occurrence of headaches and dizziness severely impacts patients' physical and mental health, resulting in a marked decline in quality of life, work productivity, and psychological well‐being [5, 6]. Furthermore, VM is often mistaken for peripheral vestibular disorders, including Ménière's disease and benign paroxysmal positional vertigo, leading to high rates of misdiagnosis and missed diagnosis. These diagnostic challenges delay appropriate treatment, prolonging patient suffering [5, 7]. The pathogenesis of VM remains unclear, with several proposed hypotheses, including genetic susceptibility, cortical spreading depression, trigeminal vascular dysfunction, central signal integration abnormalities, immune‐related factors, and ion channel dysfunction [7, 8, 9]. However, none of these hypotheses fully explain VM, highlighting the need for further research to elucidate its mechanisms, identify molecular biomarkers for early screening, and explore novel drug targets for early diagnosis and treatment.

C‐X‐C motif chemokine ligand 10 (CXCL10), also known as interferon‐gamma inducible protein‐10 (IP‐10), belongs to the CXC chemokine family and contains four conserved cysteine residues [10]. CXCL10 is a classical pain‐related chemokine, including cancer pain [11], rheumatoid arthritis‐related pain [12], chronic pelvic pain [13], pain after traumatic spinal cord injury [14], systemic lupus erythematosus‐related headache [15], and cluster headache [16]. Its level is positively correlated with the pain level. Notably, elevated levels of CXCL10 were also observed in vestibular migraine patients [17], suggesting that CXCL10 may be involved in VM development. Previous studies have demonstrated that CXCL10 activates the phosphatidylinositol 4,5‐bisphosphate 3‐kinase (PI3K)/protein kinase B (AKT) signaling pathway via C‐X‐C motif chemokine receptor 3 (CXCR3) binding, enhancing neuronal inflammation and excitability, which contributes to the maintenance of neuropathic pain [18, 19]. However, its role in VM remains unclear. This study was conducted in VM patients and rat models. The relationship between serum CXCL10 levels and the severity of headache and dizziness was analyzed, and the potential mechanism of CXCL10 in VM was explored.

## 2 Methods

### 2.1 Serum CXCL10 Expression Level of VM Patients

This study adhered to the principles of the Helsinki Declaration and received approval from the Ethics Committee of the First Hospital of Shanxi Medical University (KYLL‐2024‐129). VM patients who consulted the Headache Center between May and December 2024 were included in the study. The inclusion criteria were as follows [20]: (1) meeting the International Classification of Headache Disorders Third Version diagnostic criteria for VM; (2) aged 18–50 years, male or female; (3) providing written informed consent; (4) ability to cooperate with the assessment of relevant scales. The exclusion criteria were: (1) pregnant or lactating women; (2) presence of other vestibular disorders; (3) serious comorbidities, such as severe cognitive impairment or psychiatric disorders; and (4) current use of medications for VM, such as vestibular suppressants, migraine medications, or painkillers. Age‐ and sex‐matched healthy controls were also recruited with the following inclusion criteria: (1) no history of headaches or vestibular dysfunction; (2) no history of neurological or psychiatric disorders; and (3) ability to cooperate with blood draw procedures.

All VM patients were required to provide detailed medical histories, including general information, medical, family, and medication histories, age of onset, attack frequency, severity and duration of dizziness and headaches, and accompanying symptoms. Neurological examinations were performed, and comprehensive brain MRI and vestibular function tests were conducted. Additionally, the Dizziness Handicap Inventory (DHI) and Migraine Disability Assessment Questionnaire (MIDAS) were completed to assess vestibular disorder and migraine disability, respectively [21, 22].

Peripheral venous blood samples (3–5 mL) were collected from VM patients and healthy controls using blood collection tubes containing inert separation gel and coagulant. For VM patients, blood was collected during an interictal period on the day of sample collection. After resting at room temperature for 30 min, the samples were centrifuged at 1500× *g* for 10 min, and the supernatant was transferred into labeled cryovials and stored at −80°C until further analysis. Serum CXCL10 expression levels were measured using human interferon‐inducible protein 10 ELISA kits (Bioswamp, China). For each sample, we used triplicate wells, and the final value was calculated as the average of these triplicates to improve accuracy and reduce variability in the results. Data were analyzed using GraphPad Prism 9.0.0. Continuous variables were presented as mean ± standard deviation and median (Q1–Q3) values, and categorical variables were presented as numbers and percentages. Group comparisons were conducted using the chi‐squared test for categorical variables, the independent samples *t*‐test for normally distributed continuous variables, and the Mann–Whitney *U* test for non‐normally distributed continuous variables. Pearson correlation analysis was initially used to evaluate the relationship between CXCL10 levels and the severity of headache and dizziness. Multiple linear regression analysis was then conducted to control for potential confounding factors, including age, disease duration, and attack frequency. A significance level of *p* < 0.05 was considered statistically significant.

### 2.2 VM Rat Model Preparation

Rats were obtained from the Experimental Animal Center of Shanxi Medical University and housed in a controlled environment with regulated humidity, temperature, a regular light/dark cycle, and ad libitum access to food and water. This experiment was approved by the Ethics Committee of the First Hospital of Shanxi Medical University. Thirty‐six male Sprague–Dawley rats weighing 180–220 g were randomly divided into three groups: control group, vehicle group, and VM group, with 12 rats per group. The VM model was prepared using the methods of previous studies as follows [23, 24]: Nitroglycerin (NTG, Beijing Yimin, China) with an original concentration of 5 mg/mL was diluted to 1 mg/mL in 0.9% saline solution for use. Rats in the VM group received intraperitoneal injections of NTG (10 mg/kg) on days 1, 3, 5, 7, and 9. On day 10, rats were anesthetized with 3% isoflurane (RWD, China), and the concentration was maintained at 1.5%–2% using a mask. Kainic acid (KA, Abcam, UK) (12.5 mM, 10 μL) was injected into the bilateral tympanic cavities with rats positioned laterally. After each injection, the rats remained in the lateral position for 15–20 min. The vehicle group received intraperitoneal and bilateral tympanic injections of 0.9% saline solution, following the same protocol as the VM group. The control group underwent no interventions.

### 2.3 Behavioral Assessment

The time spent scratching and grooming within 20 min, starting 1.5 h after drug injection, was recorded for each rat (following a 20‐min adaptation period in the cage). Mechanical pain thresholds in the paws and periorbital regions were evaluated using von Frey filaments, following established protocols [25]. Baseline thresholds were measured 15–20 min before drug administration, and measurements were repeated 2 h post‐administration. Vestibular function was assessed 2 h after bilateral cochlear injections using a scoring method based on six parameters [26, 27]. Each parameter was rated on a scale from 0 to 4 (0 = normal, 4 = severe dysfunction), with a maximum score of 24. A higher score indicates greater vestibular dysfunction.

### 2.4 Western Blot

Following vestibular function assessment on day 10, the rats were euthanized by intraperitoneal injection of sodium pentobarbital (overdose dose: 100 mg/kg). Brain tissues were carefully dissected from six rats in each group. Trigeminal nucleus caudalis (TNC) and vestibular nuclei (VN) were isolated, weighed, and homogenized in enhanced RIPA lysis buffer (Boster, China) containing phosphatase and protease inhibitors (Boster, China). The TNC and VN isolation was followed by the “The Rat Brain In Stereotaxic Coordinates, 6th Edition” [28]. The TNC was sampled 14.28–15.96 mm posterior to the bregma, 7.2–9.2 mm beneath the dura mater, and 1.6–3.4 mm lateral to the midline. The VN was sampled 10–12 mm posterior to the bregma, 7–8 mm beneath the dura mater, and 1–3 mm lateral to the midline. The homogenates were centrifuged at 12,000 rpm for 15 min at 4°C, and the supernatants were collected as protein samples. Protein concentrations were determined using a BCA protein assay kit (Boster, China). Samples were subjected to SDS‐PAGE with a 10% separating gel and 5% stacking gel. Proteins were transferred onto membranes using the wet transfer method and blocked with 5% skim milk powder and 5% BSA (Boster, China). Primary antibodies, including rabbit polyclonal anti‐CXCL10 (1:1000, Proteintech, Cat#10937‐1‐AP, China), rabbit polyclonal anti‐CXCR3 (1:500, Boster, Cat#PB9079, China), mouse monoclonal anti‐AKT (1:5000, Proteintech, Cat#60203‐2‐Ig, China), mouse monoclonal anti‐p‐AKT (1:5000, Proteintech, Cat#66444‐1‐Ig, China), rabbit monoclonal anti‐PI3K (1:1000, Abclonal, Cat#A11402, China), rabbit polyclonal anti‐p‐PI3K (1:1000, Zenbio, Cat#690398, China), rabbit polyclonal anti‐interleukin‐1β (IL‐1β, 1:1000, Proteintech, Cat#26048‐1‐AP, China), rabbit polyclonal anti‐interleukin‐6 (IL‐6, 1:500, Abclonal, Cat#A0286, China), rabbit polyclonal anti‐tumor necrosis factor‐α (TNF‐α, 1:1000, Abclonal, Cat#A23264, China), and rabbit monoclonal anti‐GAPDH (1:5000, Boster, Cat#BM3874, China), were incubated with the membranes overnight at 4°C. After washing with TBST, membranes were incubated with HRP‐conjugated secondary antibodies (1:5000, Boster, Cat#BA1054, BA1050, China) for 90 min at room temperature. Protein bands were visualized using ECL substrate (Boster, China) and quantified using ImageJ software (National Institutes of Health, USA).

### 2.5 Immunofluorescence

Six rats from each group were anesthetized again following the vestibular function assessment on day 10. They were perfused with 0.9% saline solution at 4°C, followed by 4% paraformaldehyde (Servicebio, China) via intracardiac perfusion until they exhibited limb stiffness and ceased breathing. After perfusion, brain tissues were carefully dissected and fixed in 4% paraformaldehyde for 24 h at 4°C. The fixed tissues were then dehydrated in a graded series of sucrose solutions (15% and 30%) and embedded in an optimal cutting temperature compound (Sakura, USA). Brain sections (12‐μm thick) were cut using a cryostat, incubated in 0.3% Triton X‐100 (Beyotime, China) for membrane permeabilization, and blocked with 5% BSA (Solarbio, China). Sections were incubated overnight at 4°C with primary antibodies, including rabbit polyclonal anti‐CXCL10 (1:100, Proteintech, Cat#10937‐1‐AP, China), rabbit monoclonal anti‐CXCR3 (1:100, Nature Biosciences, Cat#A85330, China), rabbit monoclonal anti‐p‐AKT (1:100, Nature Biosciences, Cat#A96286, China), rabbit polyclonal anti‐IL‐6 (1:100, Abclonal, Cat#A0286, China), rabbit polyclonal anti‐IL‐1β (1:100, Proteintech, Cat#26048‐1‐AP, China), rabbit polyclonal anti‐TNF‐α (1:100, Med Chem Express, Cat#HY‐P80914, China), mouse monoclonal anti‐Iba‐1 (1:100, Abcam, Cat#ab283319, UK), mouse monoclonal anti‐GFAP (1:100, Santa Cruz, Cat#sc‐33673, USA), and mouse monoclonal anti‐NeuN (1:100, Huabio, Cat#HA601111, China). After washing, the sections were incubated with Alexa Fluor 488‐labeled goat anti‐mouse IgG (H + L) (1:500, Beyotime, Cat#A0453, China) and Alexa Fluor 555‐labeled donkey anti‐rabbit IgG (H + L) (1:500, Beyotime, Cat#A0428, China) secondary antibodies for 1 h at room temperature. The negative control was performed by omitting the primary antibody and staining only with the secondary antibody. Nuclei were stained with DAPI (Beyotime, China), and images were captured using a fluorescence microscope (Olympus, Japan).

### 2.6 Inhibition of the PI3K/AKT Signaling Pathway

To examine the role of the PI3K/AKT signaling pathway in the development of VM, the specific PI3K inhibitor LY294002 (Aladdin, Cat#154447–36‐6, China) was used for intervention. Thirty‐six male Sprague–Dawley rats weighing 180–220 g were randomly divided into three groups: VM group, VM + vehicle group, and VM + LY294002 group, with 12 rats in each group. All rats were subjected to VM modeling as described above. Rats in the VM + LY294002 group received intraperitoneal injections of LY294002 (20 mg/kg) 1 h prior to each NTG injection. LY294002 was dissolved in 0.1% dimethyl sulfoxide (DMSO) solution. Rats in the VM + vehicle group received intraperitoneal injections of the same volume of 0.1% DMSO solution, while rats in the VM group were injected intraperitoneally with NTG only. After the intervention, behavioral assessment, Western blot, and immunofluorescence assays were performed as previously described.

### 2.7 Statistical Analysis

Data were analyzed using GraphPad Prism 9.0 (GraphPad Software Inc., USA). Normally distributed continuous data were presented as mean ± standard deviation. Repeated measures analysis of variance (ANOVA) was applied for measurements obtained at multiple time points (pain threshold and grooming time) if the data satisfied normality, homogeneity of variances, and sphericity assumptions. Single‐time‐point measurements were analyzed using one‐way ANOVA. When significant differences were detected, post hoc comparisons were performed using Sidak's test for comparisons between two groups and Dunnett's test to compare each experimental group with the control group. A *p*‐value of less than 0.05 was considered statistically significant.

## 3 Results

### 3.1 Serum CXCL10 and Headache/Dizziness Scale Assessment in VM Patients

**FIGURE 1:**
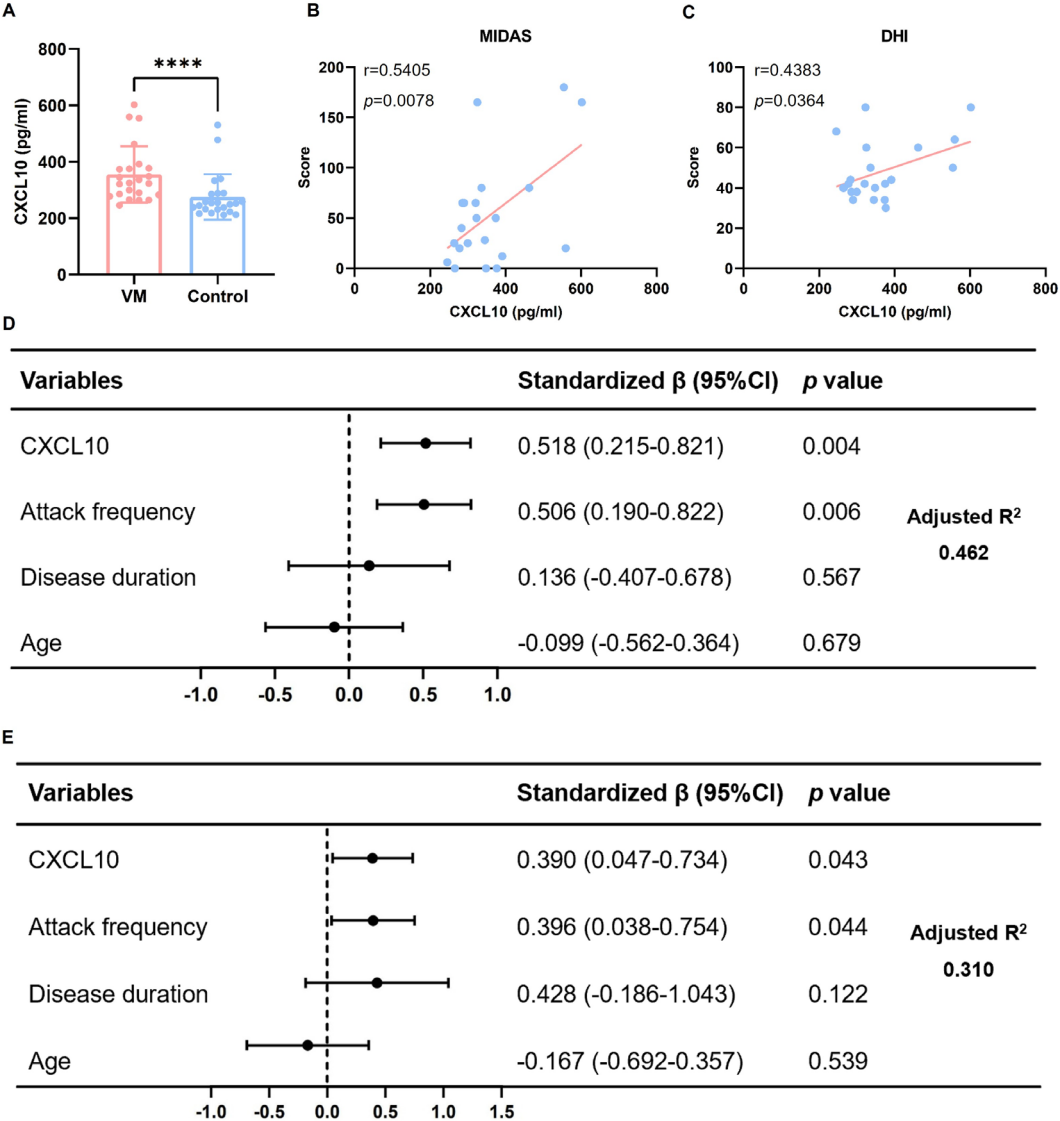
Serum CXCL10 expression and association analysis with headache and dizziness severity. (A) Serum CXCL10 levels in VM patients and healthy controls. (B) Correlation analysis between CXCL10 levels and headache severity. (C) Correlation analysis between CXCL10 levels and dizziness severity. (D) Multivariate linear regression analysis of headache severity with CXCL10, attack frequency, disease duration and age. (E) Multivariate linear regression analysis of dizziness severity with CXCL10, attack frequency, disease duration and age (*n* = 23, ****p < 0.0001).

A total of 23 VM patients and 23 healthy controls were included in this study. The demographic and clinical characteristics of the participants are presented in Table 1. There were no significant differences in age or gender distribution between the VM group and the healthy control group. Compared with the controls, serum CXCL10 levels were significantly elevated in VM patients (Table 2, Figure 1A) (Mann‐Whitney *U* test, *p* < 0.0001), confirming the hypothesis that CXCL10 may play a key role in the occurrence and development of VM. Additionally, univariate correlation analysis revealed that CXCL10 expression levels were positively correlated with both MIDAS and DHI scores (Figure 1B,C) (*r* = 0.5405, *p* = 0.0078; r = 0.4383, *p* = 0.0364). Multiple linear regression analyses were conducted with MIDAS scores (Figure 1D) and DHI scores (Figure 1E) as dependent variables, with CXCL10 level, attack frequency, disease duration, and age as independent variables. The results showed that CXCL10 level and attack frequency were significantly positively correlated with both MIDAS and DHI scores, whereas age and disease duration showed no significant association. These findings indicate that higher CXCL10 expression and more frequent attacks are associated with more severe headache and dizziness.

**TABLE 1:** Participants' demographic and clinical characteristics.

Variable		Data presentation
Gender *n* = 46	Male	21 (45.7)
	Female	25 (54.3)
Age (years) *n* = 46	Mean ± SD	25.80 ± 6.84
	Median (Q1–Q3)	25 (20.25–29)
CXCL10 (pg/mL) *n* = 46	Mean ± SD	315.32 ± 98.25
	Median (Q1–Q3)	285.57 (249.97–343.77)
Disease duration (years) *n* = 23	Mean ± SD	6.11 ± 7.99
	Median (Q1–Q3)	2 (1–10)
Attack frequency (/month) *n* = 23	Mean ± SD	9.7 ± 5.81
	Median (Q1–Q3)	10 (5–13.5)
DHI *n* = 23	Mean ± SD	47.57 ± 14.31
	Median (Q1–Q3)	42 (39–55)
MIDAS *n* = 23	Mean ± SD	51.78 ± 53.41
	Median (Q1–Q3)	40 (16–65)

**TABLE 2:** Comparison of demographics and CXCL10 levels between VM patients and controls.

Variable		Group		*p*
		VM (*n* = 23)	Control (*n* = 23)	
Gender	Male	10 (43.5)	11 (47.8)	0.767^a^
	Female	13 (56.5)	12 (52.2)	
Age (years)	Mean ± SD	25.78 ± 7.91	25.83 ± 5.76	0.495^b^
	Median (Q1–Q3)	24 (20–29.5)	25 (21–28.5)	
CXCL10 (pg/mL)	Mean ± SD	355.16 ± 97.78	281.00 ± 84.83	< 0.0001^a^
	Median (Q1–Q3)	325.10 (284.41–375.68)	252.42 (231.99–287.01)	

^a^ Mann–Whitney *U* test.

^b^ Chi‐squared test.

### 3.2 Mechanical Hyperalgesia and Vestibular Dysfunction in VM Rats

**FIGURE 2:**
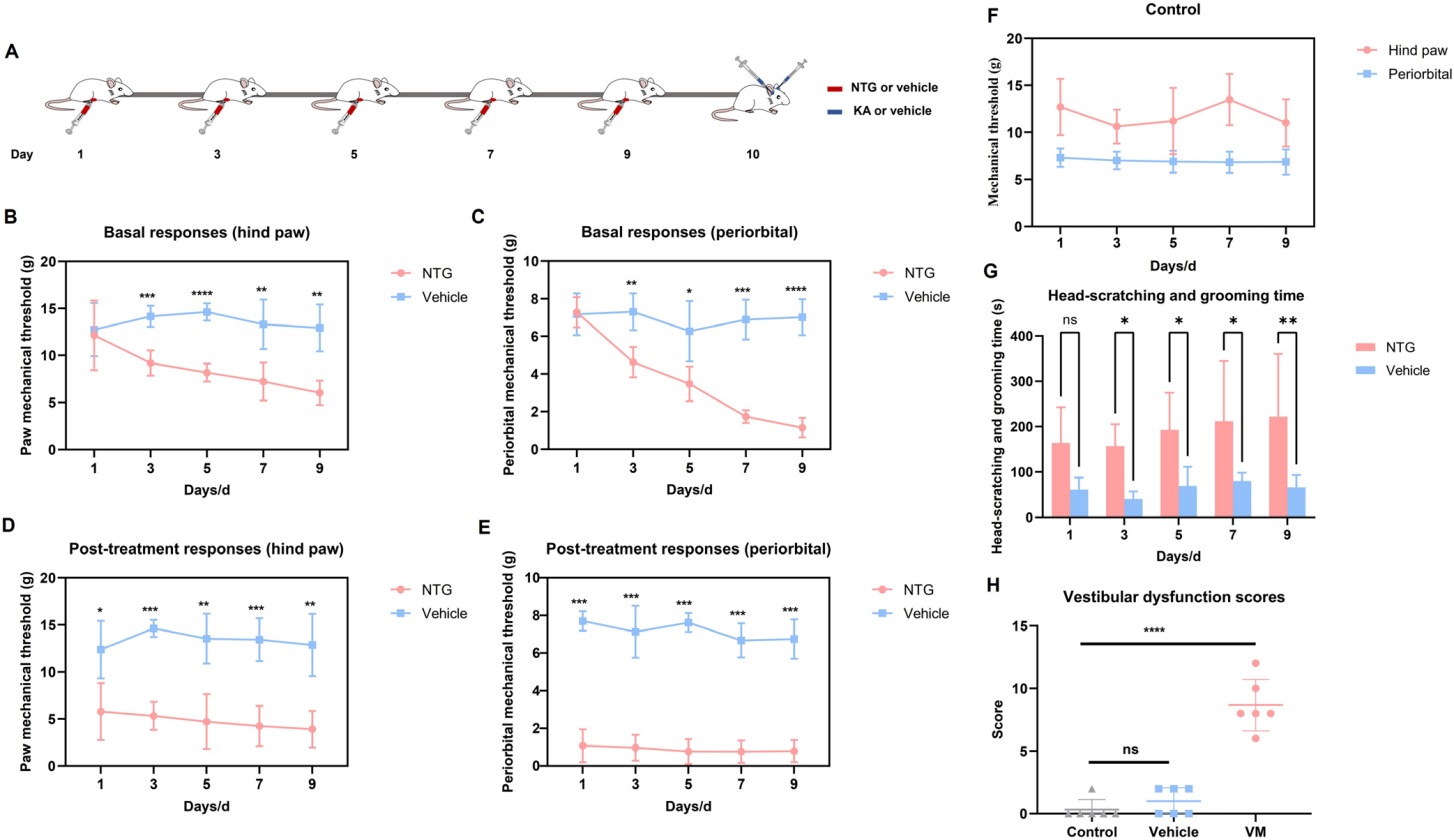
Behavioral assessment. (A) Schematic diagram of drug administration in rats. (B) Baseline mechanical thresholds of the hind paw in VM and vehicle rats on days 1, 3, 5, 7, and 9. (C) Baseline mechanical thresholds of the periorbital region in VM and vehicle rats on days 1, 3, 5, 7, and 9. (D) Hind paw mechanical thresholds in VM and vehicle rats 2 h after drug injection on days 1, 3, 5, 7, and 9. (E) Periorbital mechanical thresholds in VM and vehicle rats 2 h after drug injection on days 1, 3, 5, 7, and 9. (F) Baseline pain thresholds of hind paw and periorbital origin in control group rats. (G) Scratching time (20 min) was recorded 1.5 h after drug injection on days 1, 3, 5, 7, and 9 in VM and vehicle rats. (H) Vestibular function scores in VM and vehicle rats 2 h after drug injection (*n* = 6, *p < 0.05, **p < 0.01, ***p < 0.001, ****p < 0.0001).

Repeated NTG injections significantly reduced the hind paw and periorbital mechanical pain thresholds in rats and increased the time spent scratching and grooming (Sidak's test). Moreover, vestibular function was significantly impaired in the VM group compared to the control and vehicle groups (Dunnett's test), indicating the successful establishment of the VM rat model (Figure 2).

### 3.3 Increased CXCL10/CXCR3 Expression in TNC/VN of VM Rats

**FIGURE 3:**
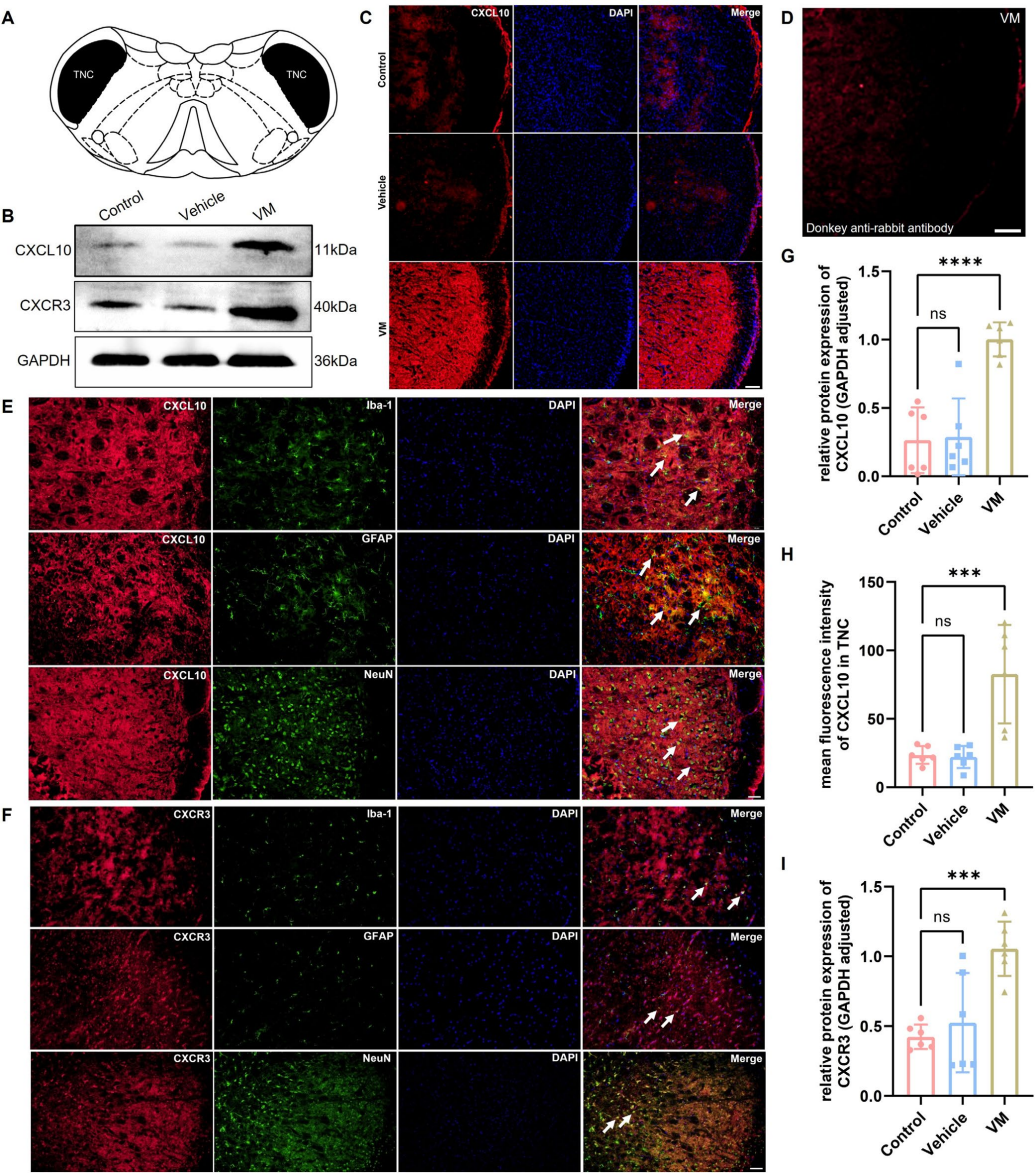
Expression level of CXCL10 and CXCR3 in TNC and co‐localization detection. (A) Schematic diagram of bilateral TNC site sampling. (B, G, and I) Western blot analysis of CXCL10 and CXCR3 expression (*n* = 6, ****p < 0.0001, ***p < 0.001). (C and H) Immunofluorescence detection of CXCL10 (Bar = 100 μm, *n* = 6, ***p < 0.001). (D) Stained with secondary antibody only (primary antibody omitted) as a negative control (Bar = 100 μm). (E and F) Immunofluorescence co‐localization of CXCL10 and CXCR3 with Iba‐1, GFAP, and NeuN. (Bar = 50 μm).

**FIGURE 4:**
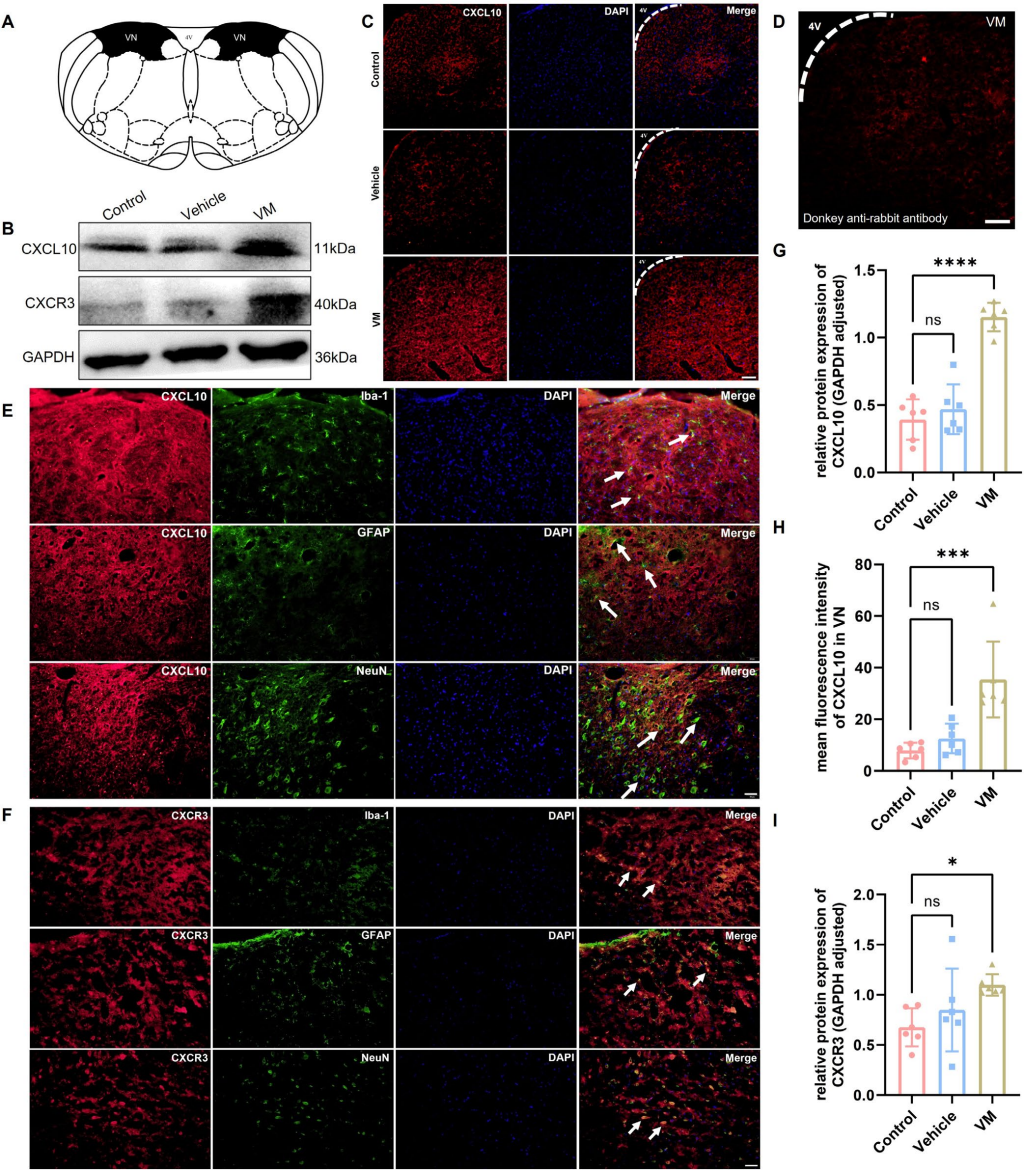
Expression level of CXCL10 and CXCR3 in VN and co‐localization detection. (A) Schematic diagram of bilateral VN site sampling. (B, G, and I) Western blot analysis of CXCL10 and CXCR3 expression (*n* = 6, ****p < 0.0001, *p < 0.05). (C and H) Immunofluorescence detection of CXCL10 (Bar = 100 μm, *n* = 6, ***p < 0.001). (D) Stained with secondary antibody only (primary antibody omitted) as a negative control (Bar = 100 μm). (E and F) Immunofluorescence co‐localization of CXCL10 and CXCR3 with Iba‐1, GFAP, and NeuN (Bar = 50 μm). 4V: fourth ventricle.

The expression levels of CXCL10/CXCR3 in TNC/VN of VM rats were analyzed using western blot and immunofluorescence. Additionally, power analysis was conducted in R using the pwr.anova.test function from the pwr package, with the significance level set at 0.05. The effect size was calculated based on data from each group. With three groups and six samples per group, the results showed a statistical power greater than 0.8, indicating that the sample size in this study was sufficient to detect differences among the groups. The sampling sites for TNC and VN are illustrated in Figures 3A and 4A. CXCL10 expression was markedly elevated in both the TNC (Dunnett's test, western blot: *p* < 0.0001; immunofluorescence: *p* = 0.0005) and VN (Dunnett's test, western blot: *p* < 0.0001; immunofluorescence: *p* = 0.0002) regions of VM rats. Consistently, the expression of its receptor CXCR3 was also increased in both the TNC (Dunnett's test, western blot: *p* = 0.0007) and VN (Dunnett's test, western blot: *p* = 0.0293) regions of VM rats. Immunofluorescence co‐localization further revealed that both CXCL10 and CXCR3 were strongly expressed in microglial cells, astrocytes, and neurons within the TNC and VN (Figures 3 and 4).

### 3.4 Activation of PI3K/AKT Signaling Pathway and Its Downstream Inflammation in TNC/VN of VM Rats

**FIGURE 5:**
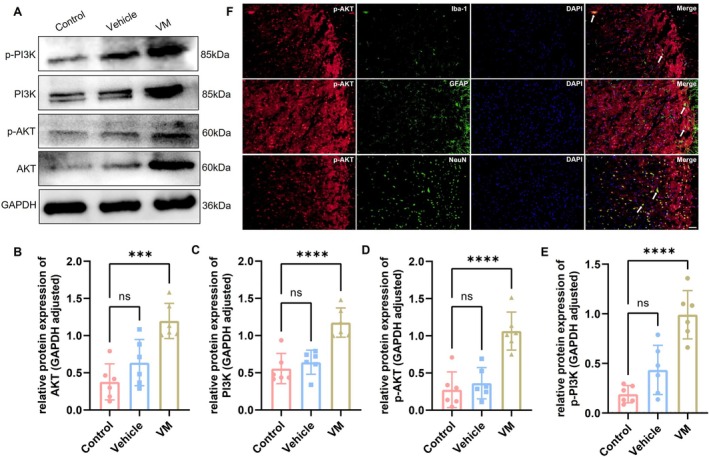
Expression level of PI3K/AKT signaling pathway in TNC and p‐AKT co‐localization. (A–E) Western blot analysis of AKT, p‐AKT, PI3K, and p‐PI3K expression (*n* = 6, ***p < 0.001, ****p < 0.001). (F) Immunofluorescence co‐localization of p‐AKT with Iba‐1, GFAP, and NeuN (Bar = 50 μm).

**FIGURE 6:**
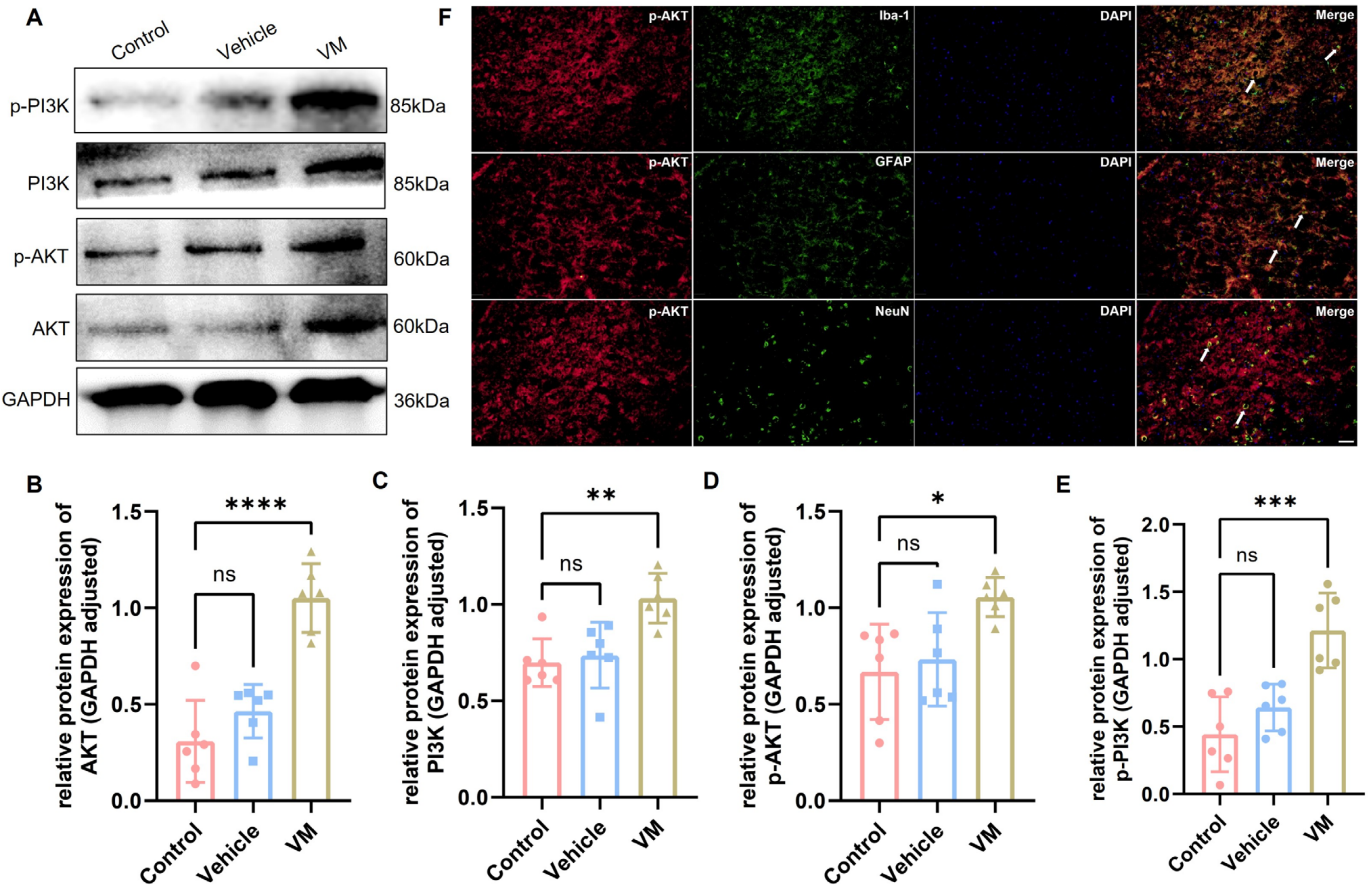
Expression level of PI3K/AKT signaling pathway in VN and p‐AKT co‐localization. (A–E) Western blot analysis of AKT, p‐AKT, PI3K, and p‐PI3K expression (*n* = 6, * *p* < 0.05, **p < 0.01, ***p < 0.001, ****p < 0.001). (F) Immunofluorescence co‐localization of p‐AKT with Iba‐1, GFAP, and NeuN (Bar = 50 μm).

**FIGURE 7:**
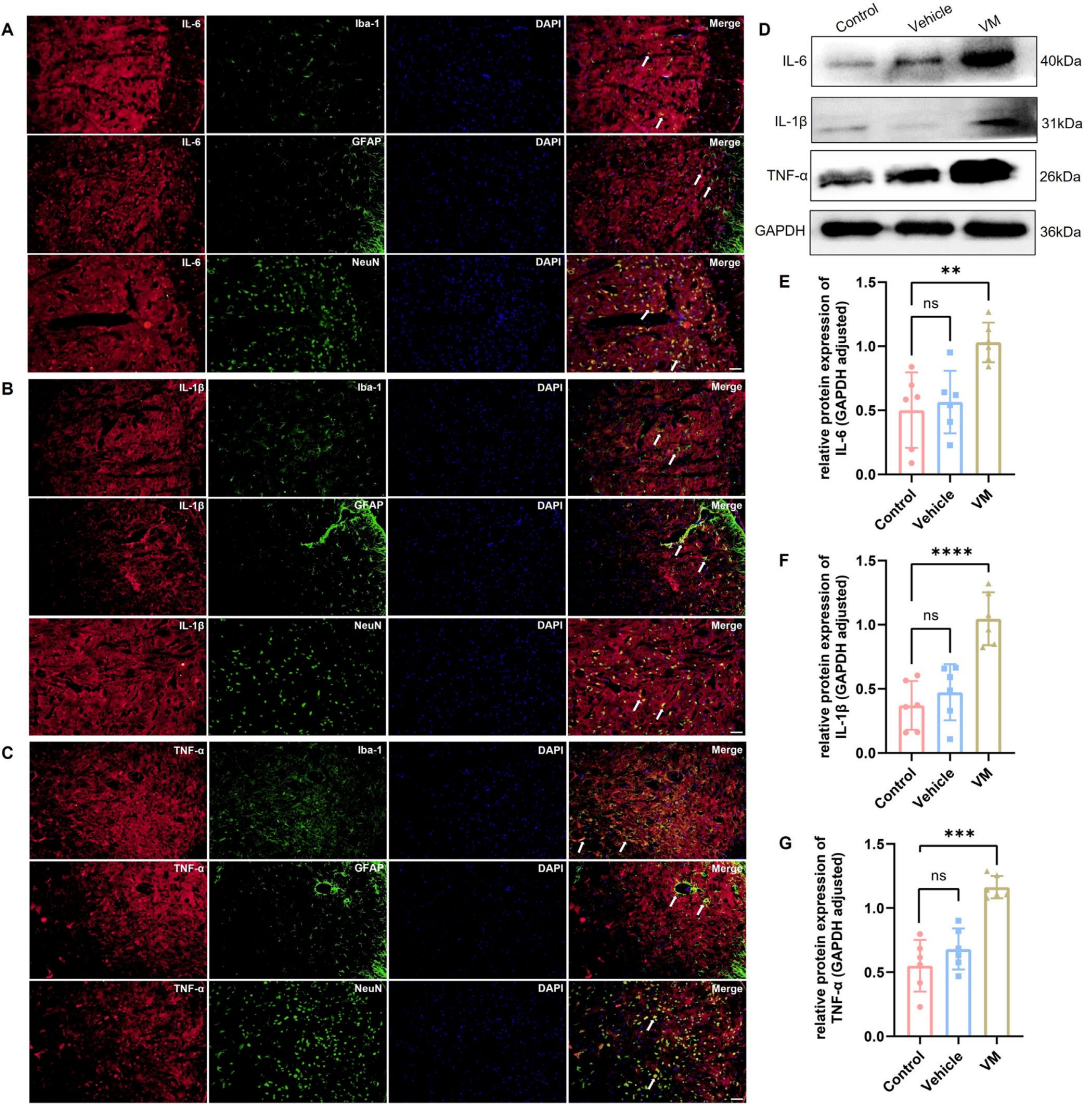
Expression level of inflammatory cytokines in TNC and co‐localization. (A–C) Immunofluorescence co‐localization of IL‐6, IL‐1β and TNF‐α with Iba‐1, GFAP, and NeuN (Bar = 50 μm). (D–G) Western blot analysis of IL‐6, IL‐1β, and TNF‐α expression (*n* = 6, **p < 0.01, ***p < 0.001, ****p < 0.001).

**FIGURE 8:**
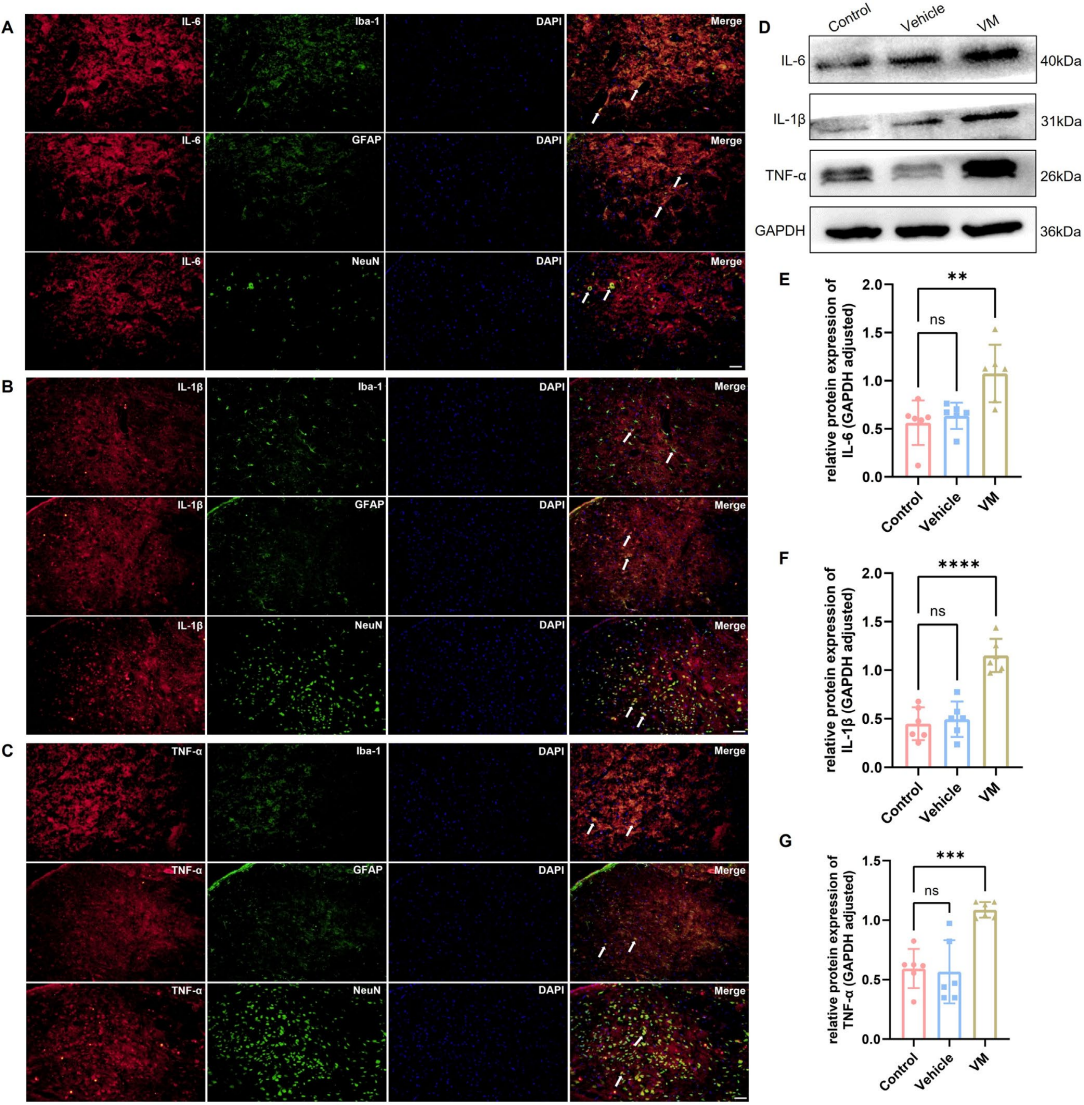
Expression level of inflammatory cytokines in VN and co‐localization. (A–C) Immunofluorescence co‐localization of IL‐6, IL‐1β and TNF‐α with Iba‐1, GFAP, and NeuN (Bar = 50 μm). (D–G) Western blot analysis of IL‐6, IL‐1β and TNF‐α expression (*n* = 6, **p < 0.01, ***p < 0.001, ****p < 0.001).

Western blot was performed to assess the activation of the PI3K/AKT signaling pathway in VM rats, with results for the TNC region shown in Figure 5 and the VN region in Figure 6. Compared with the control group, the expression levels of AKT (Dunnett's test, TNC: *p* = 0.0002; VN: *p* < 0.0001), PI3K (Dunnett's test, TNC: *p* < 0.0001; VN: *p* = 0.0020), phosphorylated AKT (p‐AKT, Dunnett's test, TNC: *p* < 0.0001; VN: *p* = 0.0106), and phosphorylated PI3K (p‐PI3K, Dunnett's test, TNC: *p* < 0.0001; VN: *p* = 0.0002) were significantly increased in VM rats. Immunofluorescence co‐localization analysis revealed that p‐AKT was expressed in microglia, astrocytes, and neurons, with the strongest signal observed in neurons, indicating that PI3K/AKT pathway activation primarily occurs in neurons in VM rats. Furthermore, downstream inflammatory factors of the PI3K/AKT pathway, including IL‐1β (Dunnett's test, TNC: *p* < 0.0001; VN: *p* < 0.0001), IL‐6 (Dunnett's test, TNC: *p* = 0.003; VN: *p* = 0.0032), and TNF‐α (Dunnett's test, TNC: *p* < 0.0001; VN: *p* = 0.0006), were also significantly upregulated. Immunofluorescence co‐localization demonstrated that these inflammatory factors were expressed in microglia, astrocytes, and neurons (TNC, Figure 7; VN, Figure 8).

### 3.5 Improvement of Hyperalgesia, Vestibular Function, and Inflammatory Response by PI3K/AKT Inhibition

**FIGURE 9:**
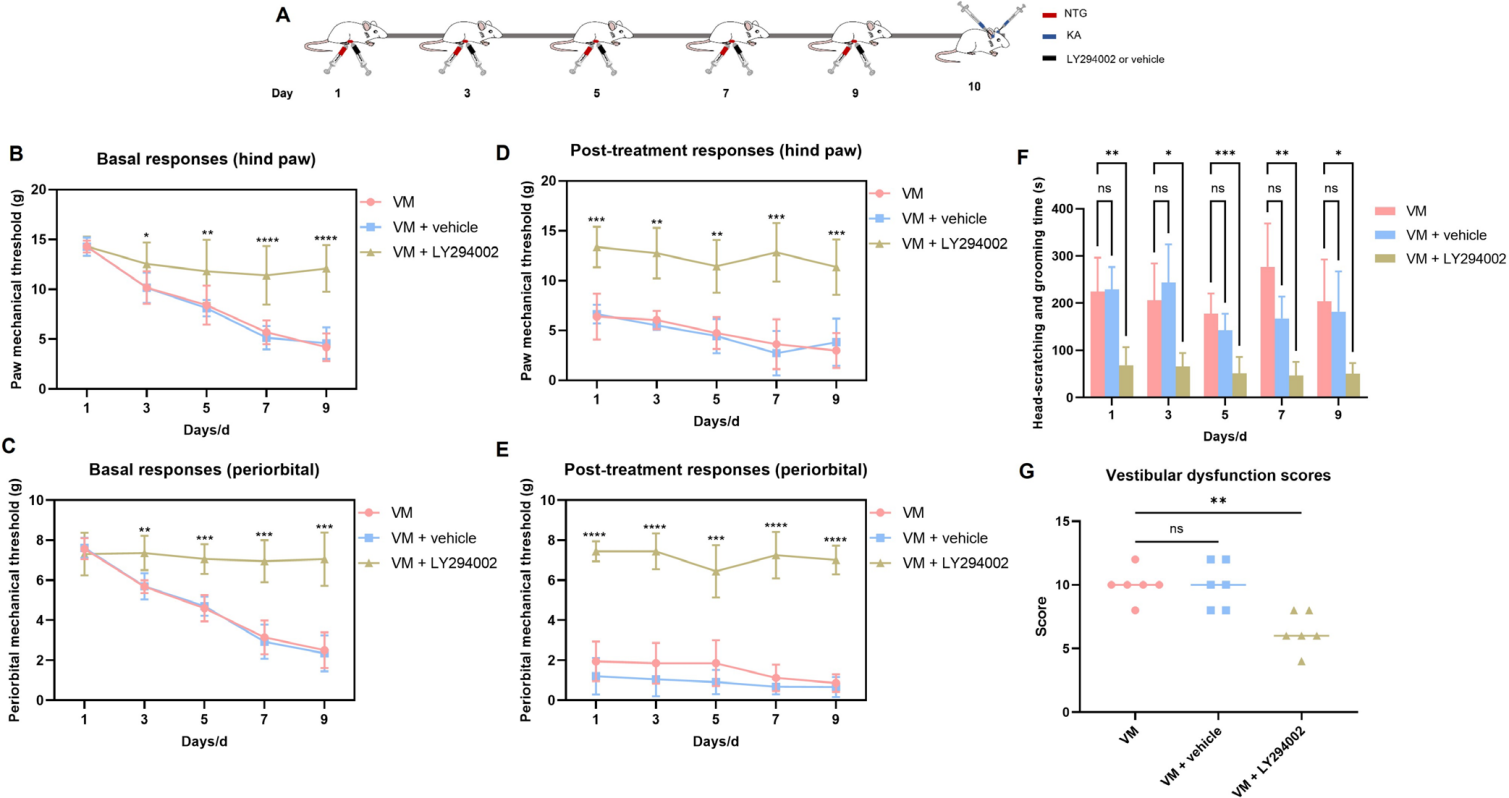
Behavioral assessment of VM rats following PI3K/AKT pathway inhibition. (A) Schematic diagram of drug administration in rats. (B) Baseline mechanical thresholds of the hind paw in rats on days 1, 3, 5, 7, and 9. (C) Baseline mechanical thresholds of the periorbital region in rats on days 1, 3, 5, 7, and 9. (D) Hind paw mechanical thresholds in rats 2 h after NTG injection on days 1, 3, 5, 7, and 9. (E) Periorbital mechanical thresholds in rats 2 h after NTG injection on days 1, 3, 5, 7, and 9. (F) Scratching time (20 min) was recorded 1.5 h after NTG injection on days 1, 3, 5, 7, and 9 in VM and vehicle rats. (G) Vestibular function scores in rats 2 h after KA injection (*n* = 6, * *p* < 0.05, **p < 0.01, ***p < 0.001, ****p < 0.0001).

**FIGURE 10:**
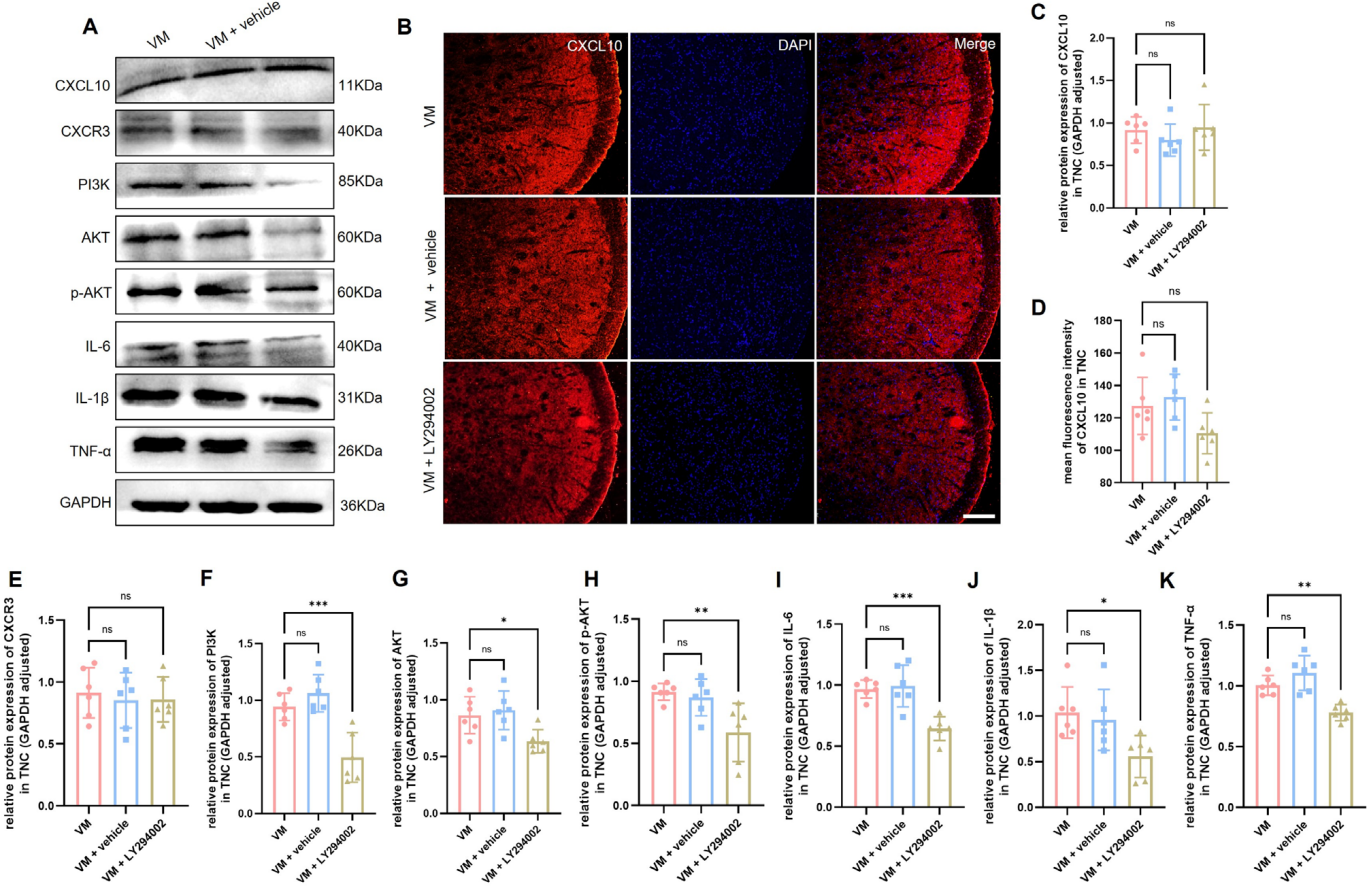
Expression level of CXCL10/CXCR3, PI3K/AKT pathway proteins, and inflammatory cytokines in TNC. (A, C, and E–K) Western blot analysis of CXCL10, CXCR3, PI3K, AKT, p‐AKT, IL‐6, IL‐1β, TNF‐α expression (*n* = 6, *p < 0.05, **p < 0.01, ***p < 0.001). (B and D) Immunofluorescence detection of CXCL10 (Bar = 100 μm, *n* = 6).

**FIGURE 11:**
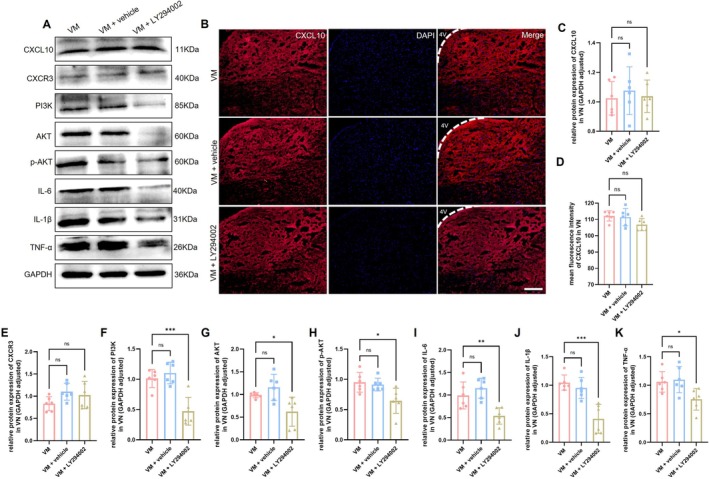
Expression level of CXCL10/CXCR3, PI3K/AKT pathway proteins, and inflammatory cytokines in VN. (A), (C) and (E) to (K) Western blot analysis of CXCL10, CXCR3, PI3K, AKT, p‐AKT, IL‐6, IL‐1β, TNF‐α expression (*n* = 6, *p < 0.05, **p < 0.01, ***p < 0.001). (B and D) Immunofluorescence detection of CXCL10 (Bar = 100 μm, *n* = 6). 4V: fourth ventricle.

LY294002 is a specific PI3K inhibitor. Behavioral assessments revealed that LY294002 significantly improved both pain sensitization and vestibular function compared to the VM and VM + vehicle groups (Figure 9). To investigate the role of the PI3K/AKT signaling pathway in VM, the expression levels of PI3K/AKT pathway‐related proteins and downstream pro‐inflammatory cytokines were detected by Western blot. As shown in Figures 10 and 11, the expression of PI3K, AKT, p‐AKT, as well as downstream cytokines IL‐1β, IL‐6, and TNF‐α, was significantly reduced in the TNC and VN of rats in the VM + LY294002 group, suggesting that the PI3K/AKT signaling pathway contributes to VM pathogenesis by regulating the expression of downstream inflammatory mediators. To further explore whether the PI3K/AKT pathway exerts regulatory effects on upstream molecules CXCL10 and its receptor CXCR3, the expression levels of CXCL10 and CXCR3 were assessed. The results showed that there were no significant differences in CXCL10 and CXCR3 expression between the VM group and VM + LY294002 group in either the TNC or VN, indicating that the PI3K/AKT pathway does not exert feedback regulation on CXCL10 or CXCR3 expression.

## 4 Discussion

VM is a complex disease characterized by recurrent episodes of vertigo associated with migraine symptoms. Due to its high prevalence and significant disability rate, research into its pathogenesis has expanded in recent years, particularly with a focus on VM biomarkers [29]. Our study demonstrated significantly elevated serum CXCL10 levels in patients with VM compared to healthy controls, a finding consistent with previous investigations. Furthermore, our analysis revealed a positive correlation between circulating CXCL10 concentrations and the severity of both headache and dizziness symptoms in the VM cohort.

Chemokines are a class of cytokines that induce cell migration and have a molecular weight of 8–10 kDa. They function by binding to seven‐transmembrane G protein‐coupled receptors on the cell membrane [30]. Based on the arrangement of two cysteine residues at their N‐terminus, chemokines are classified into four subfamilies: the CC, CXC, CX3C, and XC families. CXCL10, a member of the CXC family, exerts its effects by binding to its receptor, CXCR3 [31]. Previous basic research has demonstrated that CXCL10 plays a multifaceted role, not only in chemotaxis, differentiation, and activation of peripheral immune cells [32], but also in cell growth, apoptosis [33], and inhibition of angiogenesis [34]. Additionally, CXCL10 has been implicated in the development of pain. Studies have shown that CXCL10 expression is significantly increased in animal models of dorsal root nerve ligation, trigeminal neuralgia, and chronic nerve compression injury [18, 35, 36, 37, 38, 39]. Hyperalgesia and a reduced analgesic response to morphine were induced by intrathecal injection of recombinant CXCL10 protein in mice [19]. Furthermore, intraperitoneal injection of polyinosinic‐polycytidylic acid significantly increased CXCL10 expression in the mouse hippocampus, leading to enhanced excitatory synaptic transmission and neuronal hyperexcitability [40]. In spinal cord injury‐induced neuropathic pain, downregulation of CXCL10 expression has been shown to alleviate pain significantly [36, 38, 39]. While these studies have established the role of CXCL10 in neuropathic pain, no studies to date have explored its connection with vestibular dysfunction. However, previous research has identified fiber projections between the TNC and VN [23, 41]. Based on the elevated serum CXCL10 levels in VM patients, it is plausible to speculate that CXCL10 plays a key role in the pathogenesis of VM. Previous animal studies have demonstrated that CXCL10 is expressed in neurons, astrocytes, and microglia, with neurons showing the highest expression levels, indicating that neurons are likely the main target cells [40, 42, 43]. Consistent with these findings, our study also found that CXCL10 was mainly localized in neurons. Further studies indicate that CXCL10 contributes to the maintenance of central sensitization and neuropathic pain by activating CXCR3 [35, 40, 43]. Administration of CXCR3 antagonists or knockdown of CXCR3 expression significantly alleviates pain hypersensitivity in rats, suggesting that CXCR3 plays a critical role in pain maintenance [35, 43]. CXCR3 has been reported to be expressed in neurons, microglia, and astrocytes, predominantly in neurons [37, 44, 45]. In agreement with these reports, our study revealed that CXCL10 mainly acts in neurons through specific interaction with CXCR3, suggesting that CXCL10/CXCR3 may play a critical role in the pathophysiology of VM.

The PI3K/AKT signaling pathway is a core pathway regulating cell growth, proliferation, motility, metabolism, and survival and is involved in various physiological and pathological processes [46]. It has been reported that the PI3K/AKT pathway is activated in animal models of cancer pain, neuropathic pain, and migraine, primarily manifested by significant upregulation of AKT, PI3K, and their phosphorylated forms (p‐AKT and p‐PI3K). Activation of this pathway is closely associated with central sensitization and the maintenance of pain [47, 48, 49]. In a mouse model of trigeminal neuralgia, CXCL10 binds explicitly to CXCR3, inducing activation of the PI3K/AKT signaling pathway. This activation increases neuroinflammation and neuronal excitability, contributing to the maintenance of trigeminal neuralgia [18]. Based on these previous findings, this study examined the expression levels of PI3K, p‐PI3K, AKT, and p‐AKT in VM rats to investigate whether this pathway is activated in VM. The results showed that all these molecules were significantly elevated in VM rats, indicating that the PI3K/AKT signaling pathway is also activated in VM. Moreover, immunofluorescence co‐localization analysis revealed that p‐AKT was predominantly expressed in neurons, suggesting that the activation of this pathway mainly occurs in neurons and may play a crucial role in their function, which is consistent with previous studies [50, 51].

Inflammatory cytokines play a significant role in headache and neuropathic pain. It has been reported that activation of the PI3K/AKT signaling pathway can promote CXCL10‐induced production of inflammatory mediators [19]. In the present study, we further examined the expression levels of the inflammatory cytokines IL‐1β, IL‐6, and TNF‐α, which are downstream of the PI3K/AKT pathway. The results showed that the expression of these cytokines was significantly upregulated in VM rats, supporting the inflammatory hypothesis proposed in previous studies [52, 53]. Moreover, we observed that these cytokines were not only expressed in microglia and astrocytes but also showed prominent co‐localization signals in neurons. Previous reports have also indicated that the levels of inflammatory mediators in both glial cells and neurons are markedly increased under inflammatory conditions, playing a key role in pain maintenance [54, 55]. We speculate that CXCL10 binds explicitly to CXCR3, activating the PI3K/AKT signaling pathway and promoting the release of inflammatory factors, contributing to the pathogenesis of VM. To further validate this hypothesis, the PI3K/AKT inhibitor LY294002 was used to block the activation of this signaling pathway. The results showed that administration of LY294002 significantly ameliorated pain hypersensitivity and vestibular dysfunction in VM rats, and markedly reduced the expression of inflammatory cytokines. These findings indicate that inhibition of PI3K/AKT pathway activation effectively alleviates VM‐related clinical symptoms and inflammatory responses. This result is consistent with previous reports showing that LY294002 inhibits the activation of the PI3K/AKT pathway and significantly alleviated inflammatory responses and pain [56, 57, 58], suggesting that blocking the PI3K/AKT pathway may serve as a potential therapeutic target for VM.

There are some limitations to this study. We observed increased CXCL10 expression in VM and identified its downstream mechanisms; however, the upstream factors leading to CXCL10 upregulation remain unclear and require further investigation. In addition, whether inhibition of CXCL10 or CXCR3 could serve as a potential therapeutic strategy for VM remains to be further studied.

## 5 Conclusion

In addition to its established role in neuropathic pain, this study is the first to report that CXCL10 is involved in VM through the PI3K/AKT signaling pathway. Serum CXCL10 expression was elevated in VM patients and positively correlated with headache and dizziness severity. Furthermore, increased expression of CXCL10, its receptor CXCR3, and downstream inflammatory factors (IL‐1β, IL‐6, and TNF‐α) in the PI3K/AKT signaling pathway were observed, suggesting a key role of this pathway in VM pathogenesis.

## Author Contributions

Conceptualization: M.‐m.S. and S.‐y.X.; data curation: T.‐y.C., S.‐n.S., and Y.‐j.G.; formal analysis: M.‐m.S. and T.‐y.C.; investigation: C.‐x.L.; methodology: M.‐m.S., T.‐y.C., S.‐n.S., Y.‐j.G., and S.‐y.X.; project administration: C.‐x.L.; resources: S.‐y.X.; supervision: C.‐x.L. and S.‐y.X.; writing – original draft: M.‐m.S. and S.‐y.X.; writing – review and editing: M.‐m.S. and S.‐y.X. All authors approved the final version of the manuscript.

## Ethics Statement

This study was approved by the Ethics Committee of the First Hospital of Shanxi Medical University (Ethics Code: KYLL‐2024‐129) in May 2024. All participants provided written informed consent prior to enrolment in the study. This research was conducted ethically in accordance with the World Medical Association Declaration of Helsinki.

## Conflicts of Interest

The authors declare no conflicts of interest.

## Data Availability

The datasets generated during and/or analyzed during the current study are available from the corresponding author on reasonable request.
